# CYTO-SV-ML: A Machine Learning Tool for Cytogenetic Structural Variant Analysis in Somatic Cell Type Using Genome Sequences

**DOI:** 10.3390/life15060929

**Published:** 2025-06-09

**Authors:** Tao Zhang, Paul Auer, Stephen R. Spellman, Jing Dong, Wael Saber, Yung-Tsi Bolon

**Affiliations:** 1 CIBMTR^®^ (Center for International Blood and Marrow Transplant Research), NMDP (National Marrow Donor Program), Minneapolis, MN 55401, USA; tzhang@nmdp.org (T.Z.); sspellma@nmdp.org (S.R.S.); 2 Division of Biostatistics, Institute for Health and Equity, Medical College of Wisconsin, Milwaukee, WI 53226, USA; pauer@mcw.edu; 3 Cancer Center Biostatistics Shared Resource, Medical College of Wisconsin, Milwaukee, WI 53226, USA; 4 Medical College of Wisconsin Cancer Center, Milwaukee, WI 53226, USA; jidong@mcw.edu; 5 Division of Hematology and Oncology, Department of Medicine, Medical College of Wisconsin, Milwaukee, WI 53226, USA; 6 Linda T. and John A. Mellowes Center for Genomic Sciences and Precision Medicine, Medical College of Wisconsin, Milwaukee, WI 53226, USA; 7 CIBMTR^®^ (Center for International Blood and Marrow Transplant Research), Medical College of Wisconsin, Milwaukee, WI 53226, USA; wsaber@mcw.edu

**Keywords:** structural variants, somatic cells, cytogenetic abnormality, transplant, whole genome sequencing, machine learning

## Abstract

(1) Background: Although whole genome sequencing (WGS) has enabled the comprehensive analyses of structural variants (SVs), more accurate and efficient methods are needed to distinguish large somatic SVs (SV size ≥ 1 Mb) traditionally detected through cytogenetic testing from germline SVs. (2) Methods: A customized machine learning pipeline (CYTO-SV-ML) under Snakemake automation workflow was developed with a user interface to identify somatic cytogenetic SVs in WGS data. And this tool was applied for characterizing structural variation profiles in the whole blood of patients with myelodysplastic syndromes (MDSs). Known SVs mapped from well-established open databases were split into training and validation subsets for an AUTO-ML machine learning model in a CYTO-SV-ML pipeline. (3) Results: The benchmarking performance of the CYTO-SV-ML pipeline on somatic cytogenetic SV classification displayed an area under the receiver operating characteristic curve (AUCROC) of 0.94 for translocations and 0.92 for non-translocations, a sensitivity of 0.83 for translocations and 0.85 for non-translocations, and a specificity of 0.96 for translocations and 0.82 for non-translocations. Our method (207 somatic cytogenetic SVs) outperformed a conventional SV calling pipeline (143 somatic cytogenetic SVs) in an independent validation of clinical cytogenetic records. In addition, the CYTO-SV-ML pipeline uncovered novel somatic cytogenetic SVs in 49 (89%) of 55 patients without successful clinical cytogenetic results. (4) Conclusions: Our study demonstrates the high-performance machine learning approach of CYTO-SV-ML on benchmarking SV classification from genomic sequencing data, and further validations of novel anomalies by orthogonal methods will be essential to unlock its full clinical potential of cytogenetic diagnostics.

## 1. Introduction

Cytogenetic abnormalities are clonal chromosomal structural variations characterized in more than 50% of hematological malignancies [1] due to large genomic variants with altered chromosome numbers (e.g., hyperdiploidy, trisomy, and monosomies) and chromosome structures (e.g., translocations, inversions, and deletions). MDS is a heterogeneous hematologic stem-cell malignancy characterized by ineffective hematopoiesis, abnormal cellular maturation, and peripheral blood cytopenia with hematopoietic cell transplantation (HSCT) as the only curative treatment. And conventional cytogenetic diagnostics play an essential role in the risk stratification of patients with MDS.

Although the clinical significance of cytogenetic abnormalities has been extensively studied in MDS, conventional cytogenetic diagnostic methods are inefficient in identifying all complex aberrations [2]. For example, the main primary cytogenetic test in clinical practice, chromosome karyotyping, is time consuming and limited by low cell viability and resolution with a 5 Mb size limitation. The fluorescence in situ hybridization (FISH) technique provides resolution up to 100 kb~1 Mb but only detects specific genomic regions complementary to the designed probes [3]. Microarray-based comparative genomic hybridization (aCGH) is capable of detecting imbalanced copy number variants (CNVs) at the genomic level, but it cannot detect other cytogenetic aberrations such as translocations or inversions [4]. Notably, 10% of patients with hematological malignancies have no clinical cytogenetic information due to unsuccessful, inconsistent, or inconclusive cytogenetic results [5].

Evolving high-throughput technologies such as whole genome sequencing (WGS) are capable of systematically detecting copy number changes, structural variants, and mutations with single-nucleotide resolution [6]. A recent study demonstrated the clinical utility of WGS for diagnostic testing in patients with AML or MDS with rapid genomic profiling and greater diagnostic yield than conventional cytogenetic analysis [7]. However, the application of these technologies to identify hematological cytogenetic abnormalities could be advanced in many aspects [8,9,10,11,12]. Clinical sample source compositions can differ, for example, for bone marrow versus peripheral blood [13,14]. Second, cytogenetic variant identification in ChomoSeq is often limited by a single tool for copy number variants and structural variants; the integration of multiple SV tools with different algorithms could detect more SVs. Third, ChomoSeq only utilized part of many well-established open SV databases, such as the CytoAtlas database with curated hotspots and the 1000 Genomes data as germline background [15,16]. In addition, empirical hard cutoffs of read quality statistics are applied for SV filtering, while WGS data quality could vary with different laboratory settings. Given these technical limitations, prior canonical approaches in this space might be difficult to implement without matched normal control samples. Advanced machine learning approaches enable complicated algorithms driven by high-dimensional genomic data to infer SV identification with limited options [17,18]. However, there is no machine learning pipeline offering both functionalities: SV identification and SV classification.

**Figure 1:**
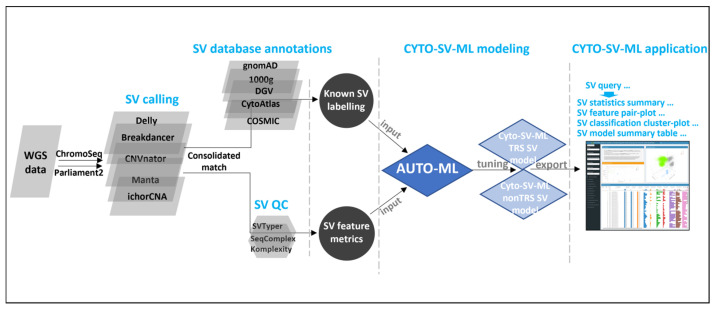
The workflow of the CYTO-SV-ML pipeline. CYTO-SV-ML pipeline workflow consists of four main modules: WGS SV calling by the integration of ChromoSeq and Parliament2 pipelines, known SV labeling by SV database annotations, SV classification modeling by AUTO-ML package, and CYTO-SV-ML interface application by dockerized R-shiny package.

In the current study, a customized SV machine learning pipeline (named “CYTO-SV-ML”) was built under Snakemake automation workflow for the optimal classification of large somatic SVs from WGS data (Figure 1). First, five SV callers were integrated for the maximum sensitivity of SV discovery, including Delly [19], CNVnator [20], Breakdancer [21], Manta [22], and ichorCNA [23]. Secondly, multiple well-established open databases were applied for known SV data labeling, including DGV [24], gnomAD [25], 1000 Genomes [15], CytoAtlas [26], and COSMIC [26]. Thirdly, the labeled SV data were fitted into a set of machine learning models for SV classification. Finally, a variety of WGS datasets of MDS patients with different clinical and laboratory settings were leveraged to demonstrate the clinical potential of our pipeline, especially in patients with failed conventional cytogenetic tests.

## 2. Materials and Methods

### 2.1. Implementation

The CYTO-SV-ML pipeline workflow is built under Snakemake automation workflow. It consists of four main modules: WGS SV calling, known SV labeling, SV classification modeling, and CYTO-SV-ML interface application (Figure 1). The source code, web portal (https://cyto-sv-ml.b12x.org, accessed on 16 August 2023) and documentation can be found at https://github.com/tzhang-nmdp/CYTO-SV-ML, accessed on 23 August 2021. The technical details can be found in the Supplementary Note.

#### 2.1.1. WGS SV Preprocessing

For WGS SV calling, five SV tools were implemented, including Delly, CNVnator, Breakdancer, Parliament2 pipeline (https://github.com/dnanexus/parliament2, accessed on 30 November 2021), Manta and ichorCNA from the ChromoSeq pipeline (https://github.com/genome/docker-basespace_chromoseq, accessed on 3 June 2021). SV genotype and quality were inferred by SVTyper (https://github.com/hall-lab/svtyper, accessed on 11 July 2021). Basic SV quality filtering criteria were applied: (1) removing SVs with an uncertain genotype; (2) removing SVs with incomplete breakpoint information, i.e., SVs must have position and confidence intervals for both ends; and (3) removing SVs with less than 1 read evidence at both breakpoints. For CYTO-SV-ML classification modeling, only SVs with length ≥ 100 kb were kept. For additional validations of clinical cytogenetic SVs, only SVs with length ≥ 1 Mb were kept.

#### 2.1.2. Known SV Labeling

For known SV labeling, four well-established open SV databases were utilized, including DGV (http://dgv.tcag.ca, accessed on 1 November 2022), gnomAD (https://gnomad.broadinstitute.org/help/sv-overview, accessed on 1 November 2022), 1000 Genomes (https://www.internationalgenome.org/phase-3-structural-variant-dataset, accessed on 1 November 2022), CytoAtlas (https://atlasgeneticsoncology.org, accessed on 1 November 2022), and COSMIC (https://cancer.sanger.ac.uk/cosmic, accessed on 1 November 2022). In the absence of matched normal DNA samples, WGS data of the corresponding transplant donors under the same lab settings were produced as sequencing technical controls. SVs from WGS data were consolidated and mapped to well-established open SV databases based on the matching criteria: (1) 90% overlapping for SV types of deletion, duplication, and inversion; (2) 1000 bp breakpoint distance for translocations. True negative artifact SVs were defined as the SVs 90% matched to gnomAD records with quality issue marks (PCRPLUS_ENRICHED, VARIABLE_ACROSS_BATCHES, PREDICTED_GENOTYPING_ARTIFACT, etc.), or high allele frequency (AF) in normal donor WGS data, or detected within the centromere region. True germline SVs were defined as SVs matched to gnomAD or 1000 genomes records that passed quality and missing true artifact SVs. True somatic cytogenetic SVs were defined as SVs matched to CytoAtlas or COSMIC records and absent in gnomAD, 1000 genomes, and normal donor WGS data.

#### 2.1.3. SV Classification Modeling

For SV classification modeling, an ensemble machine learning pipeline AutoML (https://github.com/mljar/mljar-supervised, accessed on 2 May 2021) was implemented. AutoML has 3 built-in pre-settings with cross-validations and 12 integrated machine learning models. The input features consist of 3 metrics: SV read metrics and breakpoint metrics extracted from SV tools and SVTyper, sequencing complexity metrics from SeqComplex (https://github.com/caballero/SeqComplex, accessed on 20 November 2021) and Komplexity (https://github.com/eclarke/komplexity, accessed on 20 November 2021). Two additional read features, read_diff and read_ratio, were generated (read_diff = alt_read-ref_read; read_ratio = alt_read/(alt_read + ref_read)). Overall, 90% of the labeled SV data from either small or large cohorts was used to tune AutoML models (70% of these 90% data will be applied for training and 30% of these 90% data will be applied for testing). Three layers of independent validation have been applied to our machine learning models: (1) the independent validation was conducted using the remaining 10% data that was held back from the same cohort; (2) additional independent validation was conducted using the small cohort on the model pretrained on the large cohort; (3) to further assess the clinical significance of our work, extended validation was conducted using available clinical cytogenetic information in half of the MDS patient samples. The optimal sensitivity and specificity are based on the thresholds which maximize the sum of sensitivity and specificity. Here, the Xgboost algorithm was chosen for our MDS cohorts because of its optimal performance (Table S1). Due to algorithm differences between translocation SVs and other SVs, they were trained separately. All WGS SV classification and performance metrics were produced based on the optimal trained model.

#### 2.1.4. CYTO-SV-ML Interface Application

To visualize the SV classification performance of our CYTO-SV-ML pipeline, an interactive application was created for production on a Dockerized R-shiny platform (version: 3.6.1). The classification summary for all SV records is presented on multiple featured lists and plots. In this interactive application, the user can define the genomic coordinates of input SVs and then check the model prediction probabilities and WGS data statistics in a summary textbox and 3D scatter plot. To illustrate the correlations between SV feature and SV classification, the user can check the individual SV feature distributions in a paired dot plot and a dynamic summary table for the entire cohort.

### 2.2. Biological Cohort, DNA Extraction

To evaluate the flexibility of our CYTO-SV-ML pipeline, two MDS cohorts were included with different clinical and laboratory settings (Table S2). Data from the large cohort with 494 MDS subjects were randomly selected for a cohort study processed by Broad Institute [27], while the small cohort with 94 MDS subjects was selected for a case-control study and processed by the Medical College of Wisconsin [28]. Of note, all the patients in the small cohort were wild type for *TP53*, *RAS* pathway and *JAK2* mutations, and controls were matched to cases on the following variables: Age +/−5, Regimen intensity, IPSS-R score at HCT, Donor type, Length of follow up since HCT. Overall, 588 (494 + 94) patient and 588 (494 + 94) donor samples were derived from whole blood collected in ACD-A tubes. Patient peripheral blood cell (PBC) samples were collected before the administration of the preparative conditioning regimen prior to transplantation. All samples were shipped overnight at room temperature to the Center for International Blood and Marrow Transplant Research (CIBMTR) Research Repository, aliquoted on the day of receipt and stored frozen at −80 °C or in liquid nitrogen. Samples were extracted using either the Qiagen Puregene Method or DNA Blood Kit on the Perkin Elmer Chemagic 360 (PerkinElmer, Waltham, MA, USA). DNA yield and sample concentration depend on the quantity and quality of the blood sample submitted. Generally, approximately 100–500 ng/uL gDNA was yielded using a Qiagen Puregene kit (Qiagen, Hilden, Germany). Tumor-only DNA samples were then processed with deep coverage human whole genome sequencing (WGS). The study was approved by the Institutional Review Board of CIBMTR and conducted in accordance with the Declaration of Helsinki.

### 2.3. Whole Genome Sequencing

Whole genome sequencing was performed by the Broad Institute using a modified Illumina TruSeq PCR-Free LT Library Prep protocol. Briefly, 3–4 µg of genomic DNA (gDNA) was fragmented using Covaris acoustic shearing to a target size of 900–1000 bp. The resulting fragments underwent dual size selection with AMPure XP beads (Beckman Coulter, Brea, CA, USA), first to enrich for larger fragments and then to exclude smaller ones. End repair was carried out following the standard Illumina protocol, followed by a second identical dual size selection step to further refine fragment size. Adenylation and adapter ligation were performed according to Illumina’s standard procedures. After ligation, libraries were purified using two additional rounds of AMPure bead cleanup. Library quality and concentration were assessed using a Fragment Analyzer (Advanced Analytical, Parkersburg, WV, USA) and quantified via qPCR with Universal qPCR MasterMix (Kapa Biosystems, Wilmington, MA, USA) on a CFX384 quantitative thermal cycler (Bio-Rad, Hercules, CA, USA). Libraries were then pooled at a concentration of 3 µM and initially sequenced on the Illumina MiSeq (Illumina, San Diego, CA, USA) to evaluate cluster density, yield, and index distribution. Samples were normalized to the appropriate concentration and sequenced on the Illumina NovaSeq 6000 using 2 × 150 bp paired-end reads on an S4 flow cell. A pool of 12 samples was used to achieve a minimum of 60× coverage per sample. Raw BAM files were generated using the hg38 reference genome.

## 3. Results

### 3.1. Preparation of Known SV Data of Cytogenetic Somatic SVs

Genome-wide SVs were called on a larger cohort of patients with MDS (494 subjects) and a smaller pilot cohort (94 subjects) (Figure S1a,b, see the Methods section for cohort setting). A total of 9,173,256 (large cohort) and 993,158 (small cohort) SVs were called, respectively, by the five SV tools. After the initial QC, 370,606 (large cohort) and 127,007 (small cohort) SVs passed the filtering criteria (see the Methods section). For SV classification, 277,234 (large cohort) and 77,253 (small cohort) SVs with length ≥ 1 Mb were identified. There were 323,902 (large cohort) and 31,309 (small cohort) translocations, 31,309 (large cohort) and 5904 (small cohort) deletions, 74,235 (large cohort) and 8560 (small cohort) duplications, and 102,412 (large cohort) and 8101 (small cohort) inversions (Figure S1a,b).

**Figure 2:**
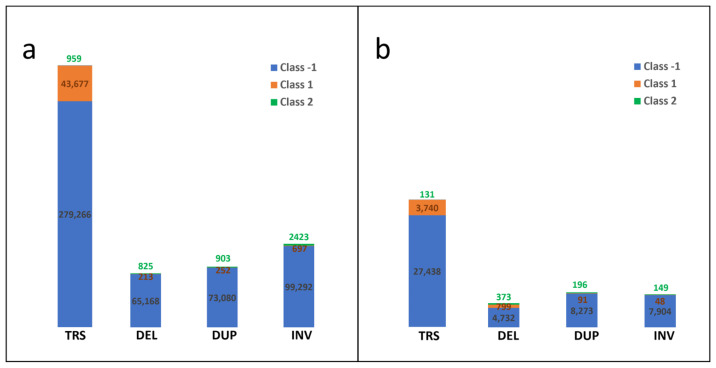
The SV subtype distributions of known SV data (SV size ≥ 1 Mb) in large (**a**) and small (**b**) MDS cohorts. Note: Class -1 is for artifact SVs; Class 1 is for true germline SVs; Class 2 is for true cytogenetic somatic SVs; TRS: translocation; DEL: deletion; DUP: duplication; INV: inversion.

To benchmark these calls, SVs were mapped to five different databases: 516,806 (large cohort) and 48,347 (small MDS cohort) SVs were labeled true negative artifact SVs, including QC failed SVs from the gnomAD database (Figure 2a,b, see the Methods section for known SV labeling). Overall, 44,839 (large cohort) and 4678 (small MDS cohort) SVs were labeled as true germline SVs, including QC passed SVs from gnomAD and 1000 genomes databases (Figure 2a,b). In addition, 5110 (large cohort) and 849 (small MDS cohort) SVs were labeled as true somatic SVs, including SVs from CytoAtlas and SVs from COSMIC databases (Figure 2a,b).

Cytogenetic information based on the karyotyping test was obtained from clinical labs for the large cohort. For the verification of SVs, potential candidates were mapped to the clinical cytogenetic records obtained prior to transplantation in 49% of MDS samples. Of the 9 translocation (TRS) and 289 non-translocation (non-TRS) SVs in clinical cytogenetic records, 6 TRS and 248 non-TRS SVs were present in our WGS SV data (Table S3).

### 3.2. The Performance of CYTO-SV-ML Pipeline

**Figure 3:**
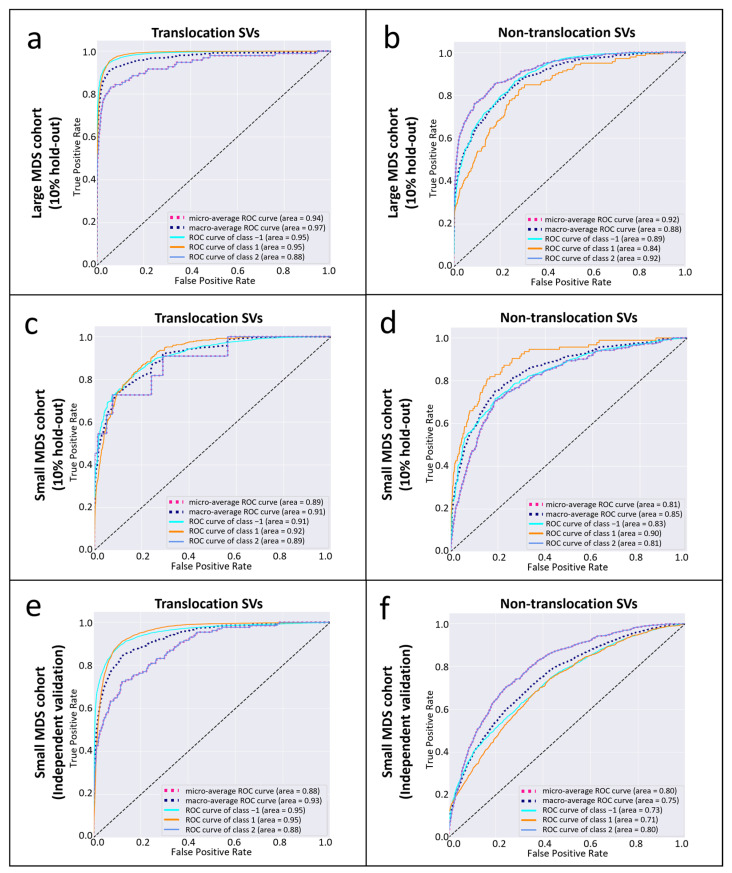
The SV classification performances of CYTO-SV-ML pipeline are robust on both large and small MDS cohorts. The AUCROC of TRS and non-TRS SV classification were assessed in large ((**a**) for TRS and (**b**) for non-TRS) and small ((**c**) for TRS and (**d**) for non-TRS) MDS cohorts using 10% hold-out validation data from the same cohorts, and small MDS cohorts as independent validation data on the models pretrained by large MDS cohort ((**e**) for TRS and (**f**) for non-TRS).

Using 10% hold-out validation data from the large MDS cohort, the optimal macro-AUC and micro-AUC for all SV classifications, and AUC for somatic SV classification were 0.94, 0.97, and 0.94, respectively, for translocation (TRS) type, and 0.88, 0.92, and 0.92, respectively, for non-translocation (non-TRS) type (Figure 3a,b, see the Methods section for the training/testing/validation data settings). The optimal sensitivity and specificity in the validation were found to be 0.83 and 0.96 for somatic translocation SVs, while they were 0.85 and 0.82 for somatic non-translocation SVs. Using the 10% hold-out validation data of the small MDS cohort, the optimal macro-AUC and micro- AUC for all SV classifications and AUC for somatic SV classification were 0.91, 0.89, and 0.89, respectively, for TRS and 0.85, 0.81, and 0.81, respectively, for non-TRS (Figure 3c,d). The optimal sensitivity and specificity in the validation were 0.72 and 0.92 for somatic translocation SVs, while they were 0.72 and 0.82 for somatic non-translocation SVs. When using the small cohort as independent validation data on the model pretrained with the large cohort, the performance is slightly lower than those using 10% hold-out data and the pretrained model from the same cohorts. Specifically, the optimal macro-AUC and micro- AUC for all SV classifications and AUC for somatic SV classification were 0.93, 0.88, and 0.88, respectively, for TRS and 0.75, 0.80, and 0.80, respectively, for non-TRS (Figure 3e,f). The optimal sensitivity and specificity in the validation were 0.72 and 0.87 for somatic translocation SVs, while they were 0.74 and 0.72 for somatic non-translocation SVs.

Using Shapley Additive Explanations (SHAP) analysis in our CYTO-SV-ML pipeline, the relative contributions of genomic and sequencing features were evaluated. For the large cohort, the read ratio was found to be the most important feature for TRS cytogenetic SV classification, while read complexity and breakpoint variations were found to be the most important features for non-TRS cytogenetic SV classification (Figure S2a). Similar patterns of feature importance were observed in the small cohort (Figure S2b).

### 3.3. Confirmation of SVs from Cytogenetic Records

Based on cytogenetic records (6 TRS and 248 non-TRS SVs present in our WGS SV data), 6 (100%) TRS and 201 (81%) non-TRS cytogenetic somatic SVs were accurately classified by our CYTO-SV-ML pipeline, whereas 6 (100%) TRS and 137 (55%) non-TRS cytogenetic somatic SVs were classified by the ChromoSeq pipeline (Table S3).

Overall, 55 (11%) patients in our cohort have missing clinical cytogenetic records prior to transplantation due to either insufficient metaphases, reduced cell viability, or other medical issues. Using WGS data, our CYTO-SV-ML pipeline discovered 196 novel somatic SVs in 49 (89%) of 55 patients. These novel cytogenetic somatic SVs need further confirmation by secondary experiments.

### 3.4. The Overview of CYTO-SV-ML Application

**Figure 4:**
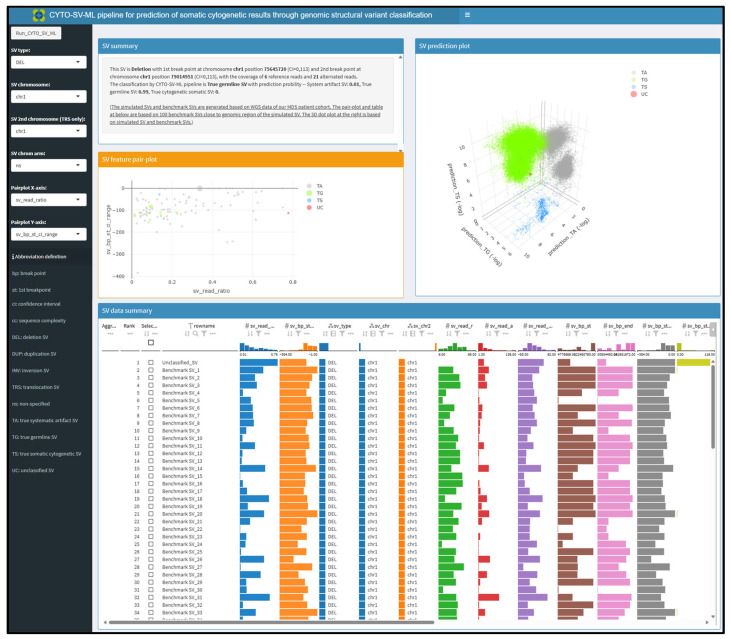
The CYTO-SV-ML web interface for the summary of cytogenetic SV classification in our MDS cohort (https://cyto-sv-ml.b12x.org, accessed on 16 August 2023). The left panel provides a set of parameters for CYTO-SV-ML, including chromosome location, SV subtype and axis variables for the SV feature plot. The right panel incorporates a textbox on the top for a summary of SV statistics, two plots in the middle, respectively, for SV feature and class distribution, and a table on the bottom for all the metrics for up to one hundred subjects simultaneously.

To provide a user-friendly interface for visualizing our results, a CYTO-SV-ML R-shiny application was built in a Docker framework (https://cyto-sv-ml.b12x.org/, accessed on 16 August 2023). Multiple layers of SV statistics on feature metrics and model prediction were integrated into this application. With WGS data from patients with MDS, SVs were plotted to illustrate the dynamic distributions of read ratio and breakpoint variation features among predicted and known SVs (Figure 4). The visualization of SV classes by 3D plots demonstrates clear clustering among predicted and known SVs (Figure 4).

### 3.5. The Runtime of CYTO-SV-ML Pipeline

Regarding the performance of the CYTO-SV-ML pipeline on WGS data (60×) using eight CPU cores, the runtime of preprocess step is 18 h (hours), including 12 h for rule run_parliment, 3 h for rule run_chromoseq, 1.6 h for rule svtyper_qc, 1.5 h for rule sv_database_ann, and 0.1 h for rest rules (see the example detail in Section S5 of the Supplementary Note).

For the CYTO-SV-ML pipeline on both MDS large and small cohorts using eight CPU cores, the runtime of the auto-ml step is 0.2 h, including 0.15 h for rule cyto_sv_ml and <0.1 h for the rest rules in the auto-ml step. The runtime of the interface step is <0.1 h.

## 4. Discussion

Capturing cytogenetic abnormality from WGS data is challenging and involves complex computational work to build a structural variation map and hierarchical clonal architecture. In the current study, a novel cytogenetic classification tool based on a machine learning approach, CYTO-SV-ML, was present with the capability to identify cytogenetic somatic SVs using WGS data. Its performance and flexibility have been validated using WGS data from different clinical and laboratory settings. Furthermore, the CYTO-SV-ML pipeline discovers more cytogenetic SVs found in clinical cytogenetic records of patients with MDS while significantly reducing labor and turnaround time compared to conventional WGS SV analytic pipelines. Lastly, it demonstrates the potential to retrieve cytogenetic information in those patients with unsuccessful conventional cytogenetic tests.

When evaluating the performances of CYTO-SV-ML and ChromoSeq with the gold standard of clinical cytogenetic records, 114 cytogenetic records were accurately classified by both pipelines. In 93 cytogenetic records only classified by CYTO-SV-ML, 69 of them were supported by ≥2 SV callers. It suggests that additional SV callers in CYTO-SV-ML confer a greater power of discovering cytogenetic somatic SVs than ChromoSeq. In 23 cytogenetic records only detected by ChromoSeq, 18 of them were classified as germline SVs by CYTO-SV-ML. It may be due to the machine learning models in the CYTO-SV-ML pipeline capturing their high similarities with structural variants present in known germline databases.

Shapley feature importance analyses showed that the read ratio was the most important feature for TRS cytogenetic SV. It might indicate that unbalanced recombination events are highly frequent for cytogenetic translocations in hematological malignancies [29], while read complexity and breakpoint variations were the most important features for non-TRS cytogenetic SVs. It might suggest that the genomic profiles of certain hot spots or regions were recapitulated for cytogenetic copy number alterations. Interestingly, feature importance analyses showed similar but not identical patterns between these two cohorts.

The key limitation of the current WGS SV study is the lack of confirmation using an orthogonal method due to the limited availability of patient pre-transplant DNA samples, especially for those additional anomaly findings which do not exist in the known SV databases or clinical cytogenetic records. The lack of a gold standard on comprehensive SV benchmarking data at the whole genome level may also be a weakness of our proposed tool CYTO-SV-ML. Whether the tool is prone to a moderate level of over-prediction or is capable of extracting additional true variations remains an open question for future benchmarking studies. One limitation of our cytogenetic validation data was the discrepancy between source samples for the clinical cytogenetic records and for the WGS data. The clinical cytogenetic records of our cohorts were mainly collected from bone marrow specimens from MDS patients, while the DNA for our WGS data was extracted from peripheral blood cells. In contrast, a recent WGS cytogenetic SV study kept consistent settings for their cytogenetic tests and whole genome sequencing by using samples from the same batches [7]. In addition, the abundance of some cytogenetic subclones was low in our WGS data derived from peripheral blood cells. These subclones may be beneath the detection threshold of WGS with 60X depth and SV calling algorithms [30,31].

The machine learning nature of the CYTO-SV-ML pipeline empowers flexibility and robustness for SV classifications but requires massive amounts of data such as whole genome sequencing data for a reliable model. When evaluating the validation performance using a 10% hold-out data set on a model pretrained with the remaining 90% from the same cohort, decent results were obtained for both the large and small cohorts. However, due to differences in clinical and experimental settings, validation of the model pretrained on the large cohort using the small cohort resulted in a slight reduction in performance. Given the high variability and complexity of genomic sequencing data, a universal modeling solution is very challenging from the analytic aspect alone. With increasing data volume and evolving technologies, the robustness of cytogenetic somatic SV classification was expected to improve.

## 5. Conclusions

Overall, the CYTO-SV-ML pipeline is a novel machine learning-based approach for benchmarking structural variant (SV) classification from genomic sequencing data, offering high flexibility and strong performance. Although further validations of novel anomalies by orthogonal methods are essential to unlock its full potential for clinical cytogenetic diagnostics, this approach highlights the promise of whole genome sequencing as a valuable alternative when conventional cytogenetic testing results are unavailable.

## Data Availability

The source code, documentation, benchmarking data and models of the CYTO-SV-ML pipeline workflow can be found on GitHub: https://github.com/tzhang-nmdp/CYTO-SV-ML, accessed on 23 August 2021 and DOI: https://doi.org/10.5281/zenodo.14640901. The web portal of the CYTO-SV-ML analytic summary can be found here: https://cyto-sv-ml.b12x.org, accessed on 16 August 2023. CIBMTR supports the accessibility of research in accord with the National Institutes of Health (NIH) Data Sharing Policy and the National Cancer Institute (NCI) Cancer Moonshot Public Access and Data Sharing Policy. CIBMTR only releases de-identified datasets that comply with all relevant global regulations regarding privacy and confidentiality. Please contact ybolon@nmdp.org for additional information.

## Supplementary Materials

The following supporting information can be downloaded at https://www.mdpi.com/article/10.3390/life15060929/s1, Figure S1: Circos plot for structural variant profiles of large (a) and small (b) MDS cohorts; Figure S2: The top important features for SV classification using the CYTO-SV-ML pipeline for the large cohort (a) and small cohort (b). Table S1: The model performance optimizations of CYTO-AUTO-ML; Table S2: The whole genome sequencing datasets for CYTO-SV-ML models; Table S3: The SV classification performance comparisons between CYTO-SV-ML and ChromoSeq pipelines on records of cytogenetic abnormalities.

## Abbreviations

The following abbreviations are used in this manuscript:

WGS	whole genome sequencing
SV	structural variation
CNV	copy number variation
MDS	myelodysplastic syndromes
ML	machine learning
AUC	area under the curve
FISH	fluorescence in situ hybridization
CMA	chromosomal microarray
COSMIC	Catalogue of Somatic Mutations in Cancer
gnomAD	Genome Aggregation Database
TRS	translocation
AF	allele frequency
PBC	peripheral blood cell
SHAP	Shapley Additive Explanations
