# Supplementary material for: CYTO-SV-ML: A Machine Learning Tool for Cytogenetic Structural Variant Analysis in Somatic Cell Type Using Genome Sequences
Source: Life (Basel). 2025 Jun 9;15(6):929. doi: 10.3390/life15060929 (PMC12194788; doi:10.3390/life15060929)
Supplement: Supplementary file 1 [file life-15-00929-s001.zip › Supplemental material MDS CYTO-SV-ML MDPI Life.pdf]

Table of Contents

Figure S1……………..2

Figure S2...3

Supplementary Note...4

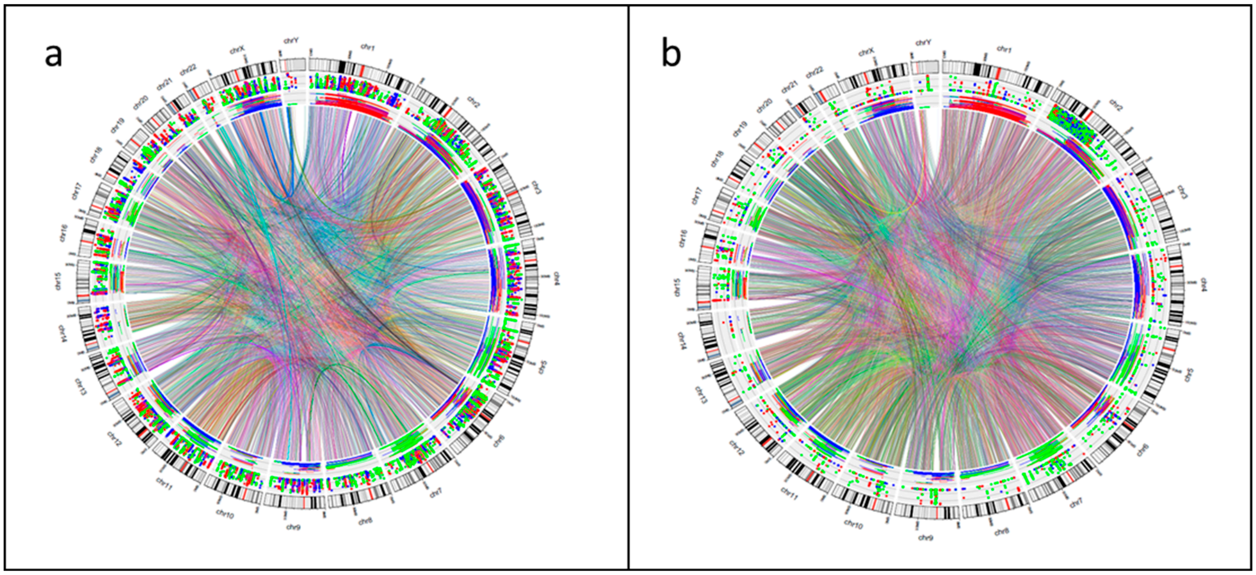

Figure S1. Circos plot for structural variant profiles of large (a) and small (b) MDS cohorts.

Note: The outer point plot is for SV break point distribution on each chromosome; the middle line plot is for nonTRS SV distribution on each chromosome; the inner link plot for TRS SV cross all chromosomes. For the outer and middle layer, red, blue, and green color, highlights duplication, deletion, and inversion SVs respectively.

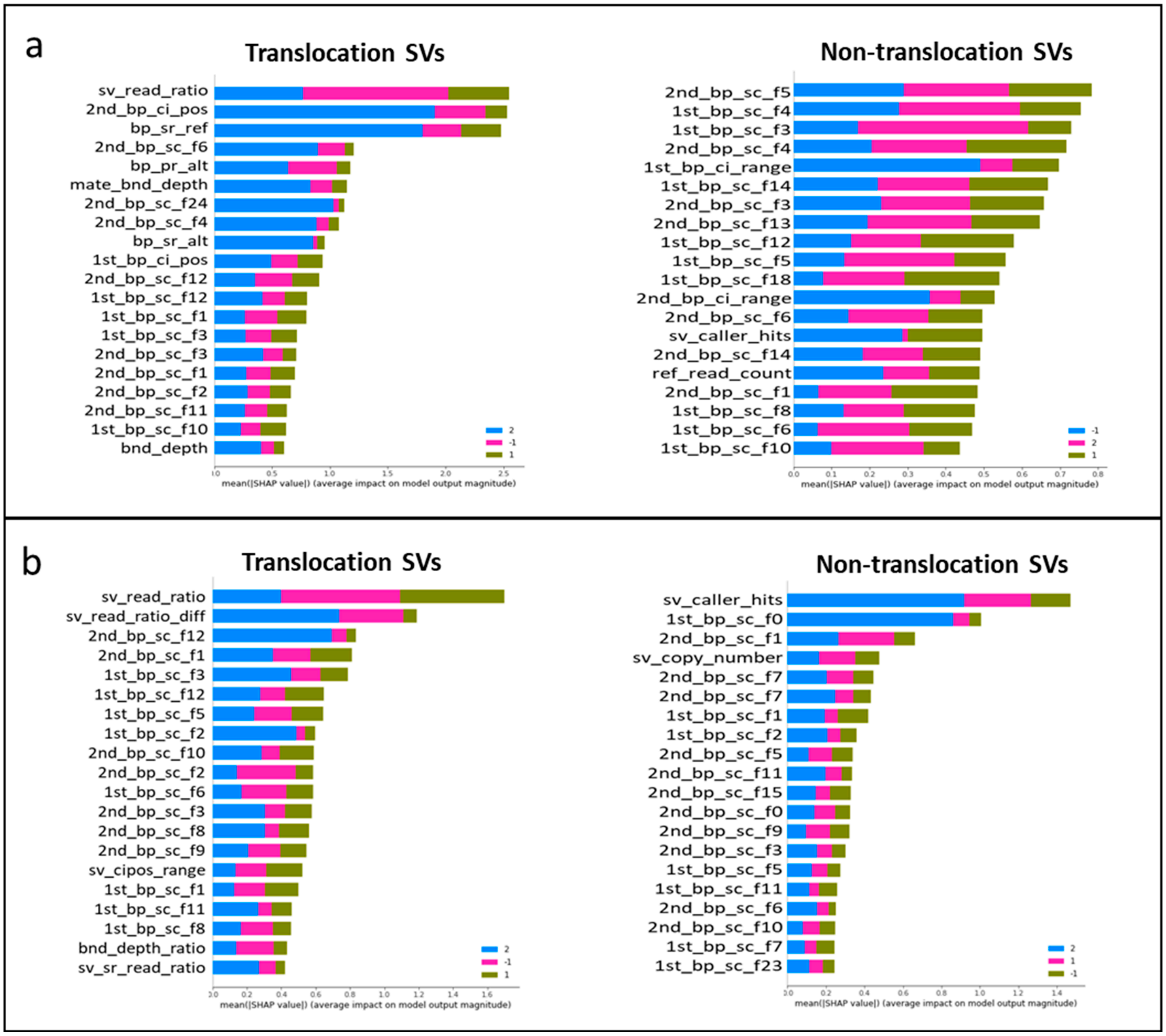

Figure S2. The top important features for SV classification using the CYTO-SV-ML pipeline for the large cohort (a) and small cohort (b).

Note: bp =break point; sc_f = sequence complexity feature; ci = confidence interval; ref = reference; alt = alternative; sr = abbreviation of split read; pr = pair read; bnd = break-end; sv =structural variant.

Supplementary Note

The CYTO-SV-ML snakemake pipeline setup

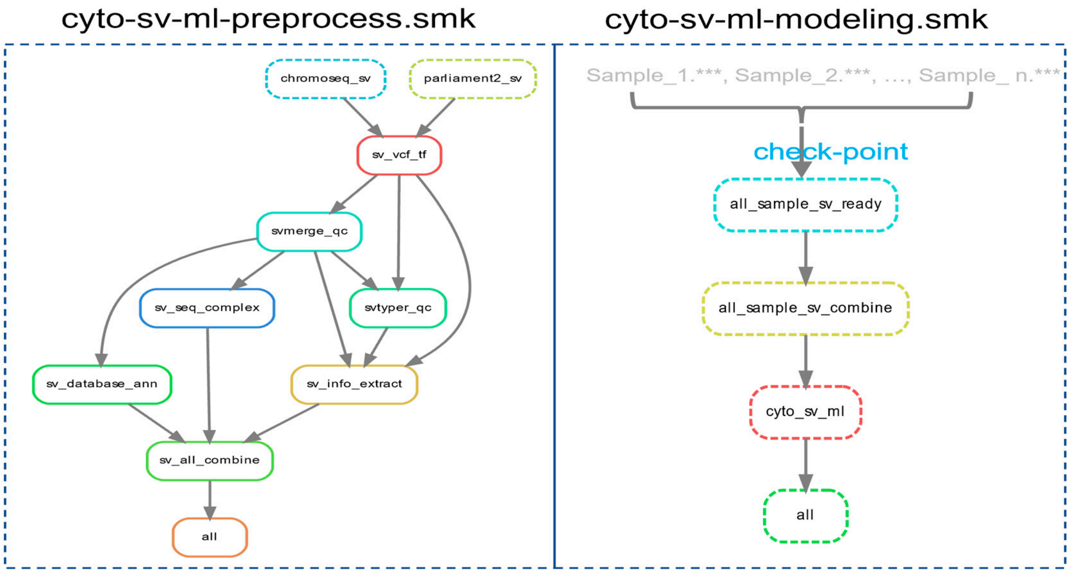

1. **Install environment**

   Conda and Docker are prerequisites and please install them according to official websites in your local environment.

2. **Download parliament and chromoseq Docker**

   # Parliament: https://github.com/dnanexus/parliament2
   sudo docker pull docker.io/dnanexus/parliament2:latest
   # ChromoSeq: https://github.com/genome/docker-basespace_chromoseq
   sudo docker pull docker.io/zatawada/docker-basespace_chromoseq_v2:master

3. **Install CYTO-SV-ML Snakemake pipeline**

   git clone https://github.com/tzhang-nmdp/CYTO-SV-ML.git
   cd CYTO-SV-ML
   mamba env create py27 -f py27.yaml
   mamba env create py39 -f py39.yaml
   mamba env create cyto-sv-ml -f cyto-sv-ml.yaml
   pip install --upgrade snakemake # in case that snakemake version is old
   # note: python-graphviz might be conflicted with the pre-existing packages in your environment. If it happened, please remove it from requirement file and install it separately.

4. **Download hg38 reference genome**

   cd CYTO-SV-ML/reference
   mkdir CYTO-SV-ML/reference/hg38
   # hg38 from Broad Institute Google Cloud

```
wget https://console.cloud.google.com/storage/browser/genomics-public-data/resources/broad/hg38/v0/Homo_sapiens_assembly38.fasta
wget https://console.cloud.google.com/storage/browser/genomics-public-data/resources/broad/hg38/v0/Homo_sapiens_assembly38.fasta.fai
mv Homo_sapiens_assembly38.fasta reference/hg38/hs38.fasta
mv Homo_sapiens_assembly38.fasta.fai reference/hg38/hs38.fasta.fai
# Please donwload VEP GRCh38 from ensembl (chromoseq pipeline)
curl -O https://ftp.ensembl.org/pub/release-90/variation/VEP/homo_sapiens_vep_90_GRCh38.tar.gz
tar xzf homo_sapiens_vep_90_GRCh38.tar.gz
cd CYTO-SV-ML/reference/homo_sapiens/90_GRCh38
```

5. Run CYTO-SV-ML Snakemake preprocess pipeline for each sample

Please change the config.yaml according to your own environment settings:

{your_work_dir} --> input/output dir
{cyto_sv_ml_dir} --> cyto_sv_ml dir and software subdir and reference subdir
{Sample_1} --> sample id
Please create input folder for chromseq pipeline --> {your_work_dir}/in/${sample}.cram
Please create input folder for parliament2 pipeline --> {your_work_dir}/in/${sample}.bam
Please create output folder --> {your_work_dir}/out/

```
conda activate cyto-sv-ml
snakemake --core ${number_of_cores} -s cyto-sv-ml-preprocess.smk --use-conda --config sample=${sample} gender=${gender}
snakemake --core ${number_of_cores} -s cyto-sv-ml-preprocess.smk --use-conda --config sample=${sample} gender=${gender} --report ${out_dir}/${ sample}/${ sample}_report.html
```

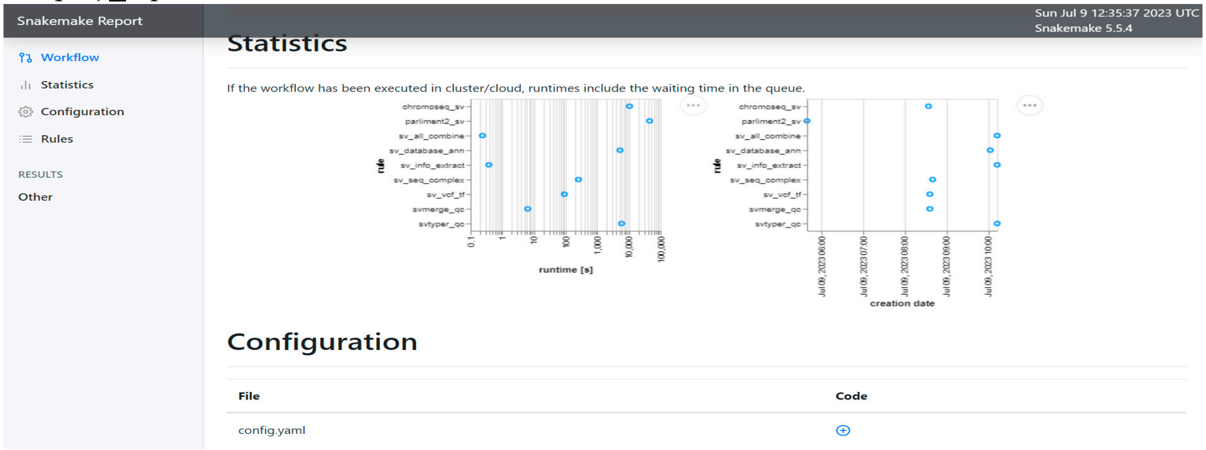

6. Run CYTO-SV-ML Snakemake modeling pipeline for whole cohort

```
snakemake --core ${number_of_cores} -s cyto-sv-ml-modeling.smk  --config cohort_name=${cohort_name}
```

snakemake --core ${number_of_cores} -s cyto-sv-ml-modeling.smk --report ${out_dir}/${cohort_name}_report.html

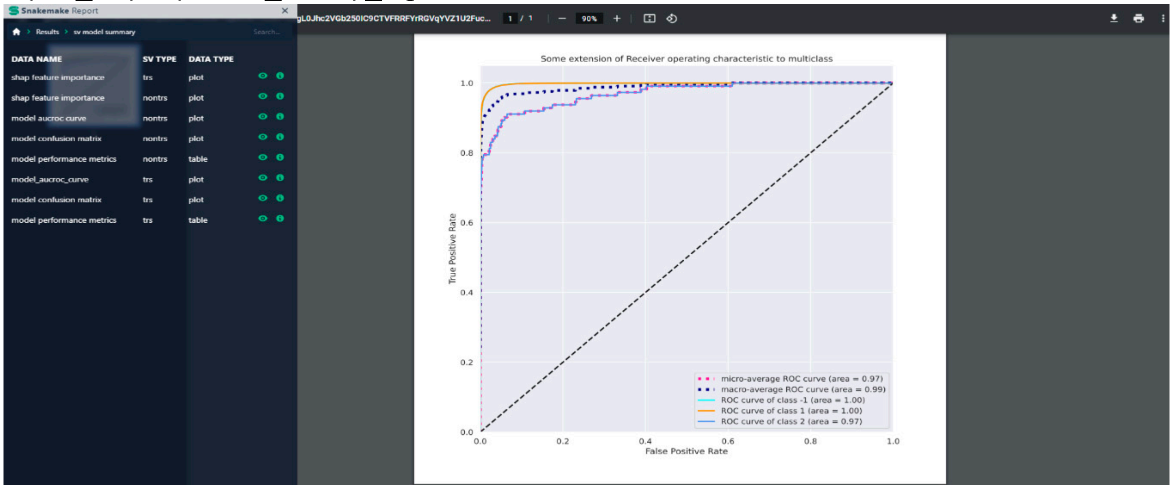

7. Run CYTO-SV-ML Snakemake interface pipeline for Web-portal application

snakemake --core ${number_of_cores} -s cyto-sv-ml-interface.smk --config cohort_name=${cohort_name} k=${kfolds}

to run the docker image in the local machine and open user interface with "http://localhost:8000/"

sudo docker run -d -p 8000:80 cyto-sv-ml-app:${sample_all}

The analysis summary of 494 MDS cohort using CYTO-SV-ML pipeline

http://cyto-sv-ml.b12x.org/

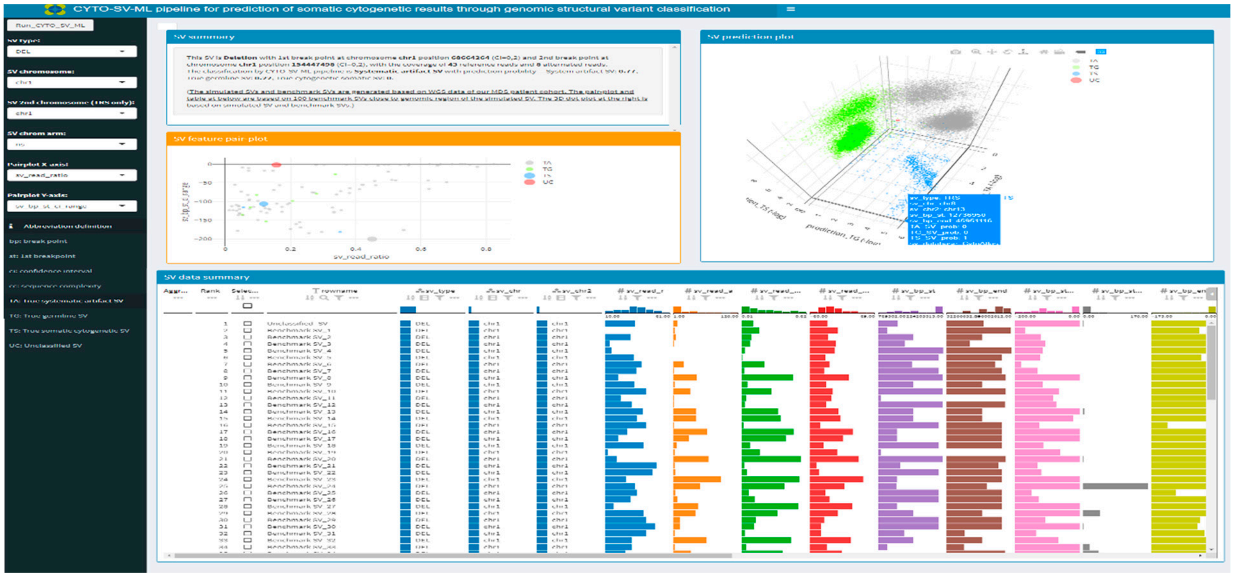

SV Related Resource Download

SV database Download (The official websites contain detailed information)

gnomAD: https://gnomad.broadinstitute.org/downloads

1000g: https://www.internationalgenome.org/phase-3-structural-variant-dataset

CytoAtlas: https://github.com/genome/docker-basespace_chromoseq/blob/master/workflow_files/chromoseq_translocations.bedpe

COSMIC: https://cancer.sanger.ac.uk/cosmic/download

SV Tool Download

SURVIVOR: https://github.com/fritzsedlazeck/SURVIVOR

SVTyper: https://github.com/hall-lab/svtyper

SeqComplex: https://github.com/caballero/SeqComplex

Komplexity: https://github.com/eclarke/komplexity

ML Pipeline Download

AUTOML: https://github.com/mljar/mljar-superviseda
